# Local and Distributed fMRI Changes Induced by 40 Hz Gamma tACS of the Bilateral Dorsolateral Prefrontal Cortex: A Pilot Study

**DOI:** 10.1155/2022/6197505

**Published:** 2022-07-16

**Authors:** Lucia Mencarelli, Lucia Monti, Sara Romanella, Francesco Neri, Giacomo Koch, Ricardo Salvador, Giulio Ruffini, Giulia Sprugnoli, Simone Rossi, Emiliano Santarnecchi

**Affiliations:** ^1^Siena Brain Investigation & Neuromodulation Lab (Si-BIN Lab), Department of Medicine, Surgery and Neuroscience, Neurology and Clinical Neurophysiology Section, University of Siena, Italy; ^2^Non-invasive Brain Stimulation Unit, Department of Behavioral and Clinical Neurology, Santa Lucia Foundation IRCCS, Rome, Italy; ^3^Unit of Neuroimaging and Neurointervention, “Santa Maria Alle Scotte” Medical Center, Siena, Italy; ^4^Neuroelectrics, Cambridge, MA, USA; ^5^Neuroelectrics, Barcelona, Spain; ^6^Human Physiology Section, Department of Medicine, Surgery and Neuroscience, University of Siena, Siena, Italy; ^7^Precision Neuromodulation Program & Network Control Laboratory, Gordon Center for Medical Imaging, Department of Radiology, Massachusetts General Hospital, Harvard Medical School, Boston, MA, USA

## Abstract

Over the past few years, the possibility of modulating fast brain oscillatory activity in the gamma (*γ*) band through transcranial alternating current stimulation (tACS) has been discussed in the context of both cognitive enhancement and therapeutic scenarios. However, the effects of tACS targeting regions outside the motor cortex, as well as its spatial specificity, are still unclear. Here, we present a concurrent tACS-fMRI block design study to characterize the impact of 40 Hz tACS applied over the left and right dorsolateral prefrontal cortex (DLPFC) in healthy subjects. Results suggest an increase in blood oxygenation level-dependent (BOLD) activity in the targeted bilateral DLPFCs, as well as in surrounding brain areas affected by stimulation according to biophysical modeling, i.e., the premotor cortex and anterior cingulate cortex (ACC). However, off-target effects were also observed, primarily involving the visual cortices, with further effects on the supplementary motor areas (SMA), left subgenual cingulate, and right superior temporal gyrus. The specificity of 40 Hz tACS over bilateral DLPFC and the possibility for network-level effects should be considered in future studies, especially in the context of recently promoted gamma-induction therapeutic protocols for neurodegenerative disorders.

## 1. Introduction

Endogenous gamma (*γ*) oscillations encompass rhythmic brain activity within the range of 35 to 100 Hz. So far, loco-regional increases in *γ* frequency have been observed in tasks such as reading and subtraction expectancy [1], as well as during memory encoding in humans and mice [2, 3], working memory [4], and chess playing [5]. However, the exact role and contribution of *γ* frequency oscillations in neural activity has been debated for a long time, with evidence supporting *γ* as a clock-like temporal framework of brain function [6, 7]. Prediction of cognitive performance looking at *γ* spectral power changes obtained through intracranial recordings—mainly in temporal and prefrontal cortices in epileptic patients—has helped to highlight the functional role of *γ* in cognition [8, 9]. Nevertheless, the neural substrates underlying such high-frequency activity are not clear yet. Early studies have suggested that *γ* oscillations result from the summed dendritic activation of pyramidal neurons in different assemblies, discharging at different rhythms [1]. On the other hand, it has been recently proposed that *γ* activity may arise from the activity of GABAergic interneurons [4], in particular parvalbumin-positive basket cells [10].

Because *γ* oscillations are involved in high-order cognitive tasks, several studies have evaluated the possibility to modulate cognitive performance in healthy and clinical populations through transcranial alternating current stimulation (tACS) [11–19], due to its ability to noninvasively influence cortical rhythms as compared to other electrical or magnetic neuromodulatory interventions [19]. However, results from previous studies are still heterogeneous. Even though tACS is considered a promising tool to study the causal relationship between oscillatory activity and brain function [11, 18, 20–24], as well as to help treating some aspects of certain neurological and psychiatric diseases [25–38], its mechanism of action and spatial specificity remain only partially understood. The online effects of tACS outside the motor cortex, as well as its target engagement specificity as predicted by biophysical modeling, are unclear and primarily based on behavioral studies. On the other hand, only the combination of tACS with neuroimaging and electrophysiological techniques can be useful to further clarify this aspect, as well as to optimally study brain connectivity and manipulate brain network activity [39, 40]. Indeed, there is a bulk of evidence showing that spectral power in *γ* frequency range correlates with an increase in blood oxygenation level-dependent (BOLD) signal [41–44] and that the correlation between local BOLD changes and local field potentials (LFP) is particularly prominent when the 25–80 Hz frequency range is considered [45].

However, the number of studies using simultaneous tACS and fMRI to specifically investigate functional MRI changes evoked by *γ* tACS is limited and challenged by potential artifacts [46–48]. Particularly, no studies investigating the BOLD signal modulation during 40 Hz tACS concurrent to fMRI recording are available so far. Moisa and collaborators have demonstrated that 70 Hz tACS enhanced motor performance which correlated with the increase of BOLD activity in the stimulated primary motor cortex (M1) [49]. On the other hand, 60 Hz tACS over Cz-Oz increased BOLD signal in frontal, parietal, temporal and occipital regions during a visual perception task, whereas higher increase of BOLD signal has been reported for 10 Hz stimulation when compared to 60 Hz and 80 Hz tACS in parietal areas at rest [50]. In all these studies, tACS effects were not limited to the target regions but rather influenced by network interactions: changes were mostly observed as modulation of internetwork functional connectivity, while intranetwork functional connectivity changes were modest [51].

Here, we present a concurrent tACS-fMRI study aimed at quantifying target engagement of 40 Hz tACS using a block design fMRI protocol. Considering the dorsolateral prefrontal cortex (DLPFC) as one of the main cortical targets in NIBS studies aimed at cognitive enhancement or clinical applications [13, 15, 26, 29, 52], we decided to stimulate this area, looking at both on-target and off-target effects. Data will allow to test whether the hypothesized 40 Hz-tACS-induced BOLD modulations are observable only at the stimulation site as predicted by biophysical modeling (on-target effects), as well as show any off-target effects relevant for planning of future tACS interventions in clinical and nonclinical populations.

## 2. Material and Methods

### 2.1. Participants

Fifteen right-handed healthy individuals (4 males and 11 females, age 26 ± 3.1), with normal neurological examination and no history of neurological or psychiatric disorders, were recruited through flyers and online advertisement. Subjects with personal and family history of epilepsy were excluded, as well as those reporting recent migraine attacks, through a self-report questionnaire. Each subject provided written informed consent. The study was approved by the Local Ethics Committee at Le Scotte Hospital and University of Siena School of Medicine (Siena, Italy; IRB protocol “APOLLO”, code: “Brainsight”).

### 2.2. Experimental Paradigm

**Figure 1:**
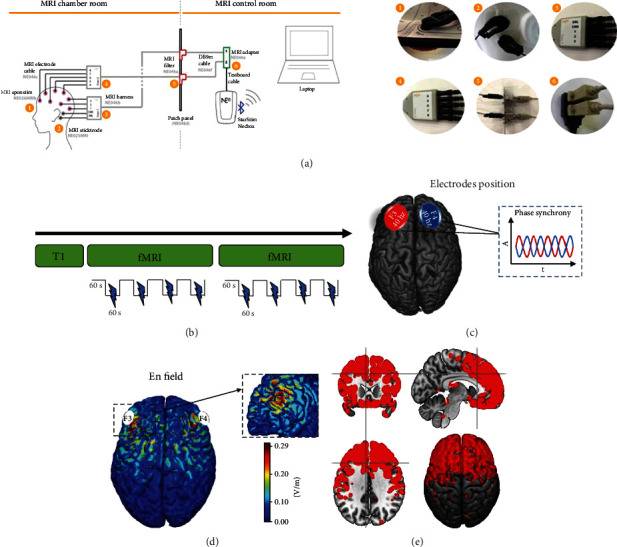
Experimental paradigm. (a) Schematic example of MRI-compatible tES device setup. (1) Details of electrode arranged in the cap, (2) CMS/DRL mastoid electrodes for impedance check, (3) and (4) MRI -compatible touchproof connector, (5) patch panel connection, (6) Starstim cable adaptor. (b) Overview of the tACS-fMRI experimental session. (c) Electrode positions and phase. (d) Normal electric field (normE) simulated on a single-subject template Colin27. (E) En binary mask thresholded at 0.8 and used as mask for the second-level analysis.

The subjects underwent a concurrent tACS-MRI protocol via an MRI-compatible stimulation system installed inside the MRI scanner (Figure 1(a)). Two fMRI runs were completed concurrently to tACS using a block design fMRI paradigm, alternating 60 seconds of 40 Hz tACS over the DLPFCs and 60 seconds of no stimulation. The experimental design is shown in Figure 1(b).

### 2.3. tACS Protocol

tACS was delivered via an MRI compatible Starstim hybrid EEG/tCS 8-channel neurostimulator system (Neuroelectrics, Barcelona, Spain). The device was connected via Bluetooth to a computer located outside the Faraday cage (Figure 1(a)). The stimulation protocol was created and monitored using the NIC 2.0 software (http://www.neuroelectrics.com/products/software/nic2/). MR-compatible electrodes (25 cm^2^) consisting of conductive rubber electrodes were used and inserted in circular sponge sockets soaked with 15 ml of sterile sodium chloride solution (0.9%) for at least 10 minutes (MRI Sponstim, Neuroelectrics). The electrodes were positioned over the right and left DLPFC (corresponding to F3-F4 in the 10/20 EEG system, Figure 1(c)) through a neoprene cap, resulting in an electric field component normal to the cortical surface (normE-field) that reached the range intensity of 0.22/0.28 V/m [53] as shown in Figure 1(d). The stimulator was connected to the MR-compatible electrodes by specially designed MR-compatible (nonferrous and radio translucent) leads.

The block design consisted of 60 seconds of no stimulation followed by 60 seconds of tACS, throughout the fMRI scan duration, for a total of 4 on blocks and 4 off blocks for each scan (Figure 1(b)). tACS was delivered as sinusoidal stimulation with no direct current offset applied at 40 Hz at an intensity of 2 mA (peak to peak). Impedances were kept below 5 k*Ω* throughout the stimulation sessions.

Prediction based on biophysical modelling suggests potential diffusion of tACS over the primary target, i.e., DLPFC, and secondary impact on additional regions affected by the specific electrode montage, such as midline frontal regions (e.g., ACC) and premotor cortex (Figure 1(e)).

### 2.4. MRI Data Acquisition

Imaging was conducted on a Siemens Avanto scanner with a 12-channel head coil (Siemens, USA). High-resolution T1-weighted anatomical images were obtained using a 3D-MPRAGE sequence (TR = 1880 ms, TE = 3.38 ms, TI = 1100 ms, flip angle (FA) = 15°, number of slices = 176, thickness = 1 mm, gap = 0 mm, imaging matrix = 256 × 256, and acquisition duration: 5 minutes). Functional MRI data were acquired before and during stimulation, using standard echo-planar BOLD imaging (TR = 2000 ms, TE = 20 ms, flip angle (FA) = 70°, number of slices = 37, thickness = 3.59 mm, gap = 4.64 mm, and acquisition duration: 8.36 minutes). Subjects were instructed not to focus their thoughts on any particular topic, do not cross their arms or legs, and keep their eyes open.

### 2.5. fMRI Data Preprocessing

fMRI data preprocessing and statistical analyses were carried out using SPM12 software (Statistical Parametric Mapping; http://www.fil.ion.ucl.ac.uk/spm/) and MATLAB 2020 (MathWorks, MA, USA) software. BOLD images underwent the following preprocessing steps: discarding of the first three volumes to allow for steady-state magnetization and stabilization of participant status, slice timing, realigning to correct for head motion; coregistration to structural images, segmentation, nonlinear normalization to the Montreal Neurological Institute (MNI) template brain, voxel resampling to an isotropic 3 × 3 × 3 mm voxel size, and smoothing with an isotropic Gaussian kernel (full width at half maximum, 8 mm). Structural images were coregistered to the mean volume of functional images and segmented using routines in SPM12. To obtain a more accurate spatial normalization, we created a customized grey matter template from all subjects' segmented images. Briefly, this approach is based on the creation of a customized anatomical template built directly from participants' T1-weighted images instead of the canonical one(s) provided by SPM (MNI template, ICBM 152, Montreal Neurological Institute). This allows a finer normalization into standard space and consequently avoids under-or overestimation of brain regions' volume. Linear trends were removed to reduce the influence of the rising temperature of the MRI scanner, and all functional volumes were bandpass-filtered at 0.01 Hz < *f* < 0.08 Hz to reduce low-frequency drifts. Finally, an important issue for brain connectivity analysis is related to the deconvolution of potential confounding signals—mainly physiological high-frequency respiratory and cardiac noise—from the grey matter voxels' BOLD time course. We decided to regress out potential confounding signals, like physiological high-frequency respiratory, cardiac noise, and all main session effects as well as the 6 rigid body head motion parameters and the signal from the CSF and white matter (WM) compartment from grey matter voxels' BOLD time course using the Compcorr algorithm [54] through an in-house code, in order to reduce artificial negative correlation and provide adequate filtering of the data.

### 2.6. Biophysical Modeling

A realistic head model based on T1-weighted and Proton Density- (PD-) weighted phantom MRI images of the single-subject template Colin27 was used to simulate the electric field distribution as previously described [55]. Five different tissue types were distinguished. Isotropic conductivities were used as follows: 0.33 Siemens per meter (S/m) for the scalp and grey matter (GM), 0.008 S/m for the skull, 1.79 S/m for the cerebrospinal fluid (CSF) (including the ventricles), and 0.15 S/m for the white matter (WM). The plugs at the apexes of the orbits were given conductivity values equal to those of the scalp. In order to represent the conductivity of sponge electrodes soaked in saline solution, the electrodes were modelled with a high conductivity value of 2 S/m. Distribution of current and normal components of the generated electrical fields is reported in Figures 1(c)–1(e) reaching the range intensity of 0.22/0.28 V/m.

### 2.7. Second-Level Analysis

Given the rationale of the study, BOLD signal changes were expected during stimulation (on blocks) under the electrodes (F3-F4) and following the topography of the En field (Figure 1(c)). Particularly, the aim of the study was to investigate target engagement during 40 Hz tACS; thus, we explored the following: (i) the impact of tACS under the targeted brain regions (F3-F4) (primary on-target effect) or in other brain areas as predicted by biophysical modelling (secondary on-target effect) and (ii) the impact of tACS at whole brain level (off-target effect). Accordingly, a Generalized Linear Model (GLM) was used to compare the images acquired during the on and off blocks, using two different approaches. Firstly, we used the En field as mask, looking at BOLD changes in regions predicted by biophysical modeling. Secondly, we performed a whole-brain voxel-wise analysis without applying any mask, looking at any off-target effects possibly provoked by tACS. Analyses were performed on both fMRI runs averaged together. Moreover, in order to investigate the functional connectivity changes induced by 40 hz tACS, we also performed a seed-to-voxel analysis. More in-depth information about the methods and results obtained are included in the supplementary material (Figure S1).

Surface representation of on/off-target BOLD changes was qualitatively mapped with the anatomical brain parcellation scheme recently published [56]. Finally, to characterize the spontaneous functional connectivity of each node, a seed-to-voxel analysis was run on a database of 1000 healthy participants [57] using the Neurosynth software.

## 3. Results

### 3.1. Participants' Experience

Participants reported common minor and transient side effects of tACS [58, 59], mostly related to tingling sensation and mild scalp burning. Approximately 90% of the participants (13 out of 15) reported phosphene perception during tACS.

### 3.2. BOLD Changes during tACS

**Figure 2:**
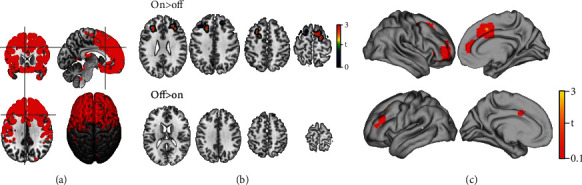
Primary and secondary on-target effect of tACS. (a) The normE-field used as mask for the second-level analysis is shown. (b) BOLD signal changes under the electrodes (DLPFC) during the stimulation, thus comparing the on blocks to the off blocks. No significant BOLD changes are shown comparing the off blocks to the on blocks. (c) A surface brain representation of the resulted nodes is shown. More details on the brain activation peaks are reported in Table 1. Right is the right side of the brain.

Considering the normE-field mask (Figure 2(a)), the comparison (two-tailed *t*-test) between on and off blocks revealed a significant BOLD increase change (height threshold *T*_(14)_ = 3.78; *p* < 0.001 uncorrected; voxel threshold: 100) during tACS (on > off) in regions located under the stimulation electrodes (primary on-target effect), such as the right and left DLPFC. We extracted the mean BOLD signal from a sphere (*r* = 10 mm) centered around the activation peak (rDLPFC: 34, 34, and 18; lDLPFC: -30, 32, and 26) for each on/off block using MarsBar toolbox (v.0.45). Within each individual's ROIs, averaged time courses comprising 8 time points (4 on blocks and 4 off blocks) were calculated in order to show the average BOLD signal change due to the stimulation. The results, fully described in the supplementary materials (Figure S2), showed a stable increase in BOLD signal along the on blocks with respect to the off ones.

A secondary on-target effect of tACS was detected in the Brodmann area 8 (BA8), a more posterior part of DLPFC that includes the frontal eye field (FEF), as well as in the anterior cingulate cortex (ACC) and premotor cortex (Figures 2(b) and 2(c), Table 1), as predicted by the biophysical modelling. On the other hand, no significant effects have been shown by the off > on contrast (Figure 2(b)).

**Figure 3:**
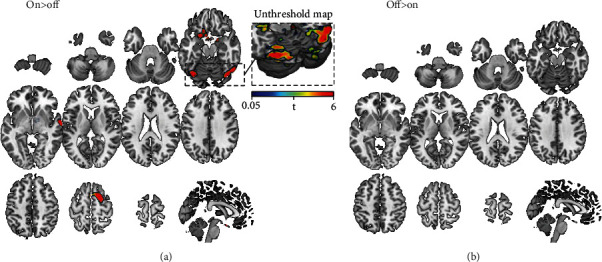
Off-target effect of tACS. (a) Significant BOLD changes that resulted from the whole brain analysis are shown in visual cortices, subgenual cortex, right temporal cortex, and SMA. (b) No significant results are shown in the absence of tACS (off > on). The results are masked for grey matter. More details on brain activation peaks are reported on Table 2. Images are presented in neurological convention (i.e., right brain is right in the figure).

Whole-brain analysis revealed significant BOLD changes (height threshold *T*_(14)_ = 1.72; *p* < 0.05 FWE corrected; voxel threshold: 100) during stimulation (on > off) in the visual cortices, subgenual cortex, right temporal cortex, and supplementary motor area (SMA) (Figure 3(a), Table 2). On the other hand, no significant differences were observed for the contrast off > on, as shown in Figure 3(b).

### 3.3. Anatomical and Functional Mapping

**Figure 4:**
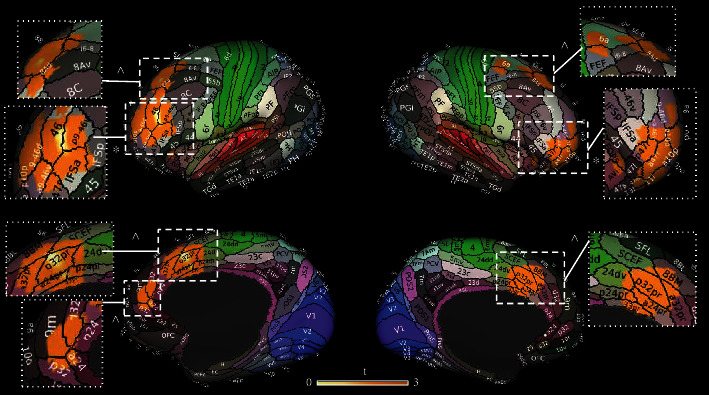
Anatomical mapping of on-target results. Qualitative overlap between the Glasser Atlas and the map of BOLD activations during 40 Hz tACS (orange) is shown. Activations on primary target bilaterally (^∗^), as well as the secondary impact on regions predicted by the biophysical modeling (^), such as the ACC and right premotor cortex, are reported.

Surface representations of BOLD signal changes have been qualitatively mapped over the anatomical brain parcellation scheme published by Glasser and collaborators. In particular, Figure 4 shows that BOLD changes within the En map overlap with DLPFC (46, p9-46, 9-46d, and IFSa), as well as with a more posterior part of DLPFC (8ad, 8bm, and 8bl) including a small part of frontal eye fields (FEF), the anterior cingulate cortex (ACC—p32r, a24pr, and 9m), and right premotor cortex (area 6a) confirming the primary and secondary on-target effect of 40 Hz tACS. As well-known, these regions are relevant for cognitive tasks, cognitive control, and planning and control motor responses [60–64]. More in-depth description of the possible role of these areas will be provided in Discussion.

**Figure 5:**
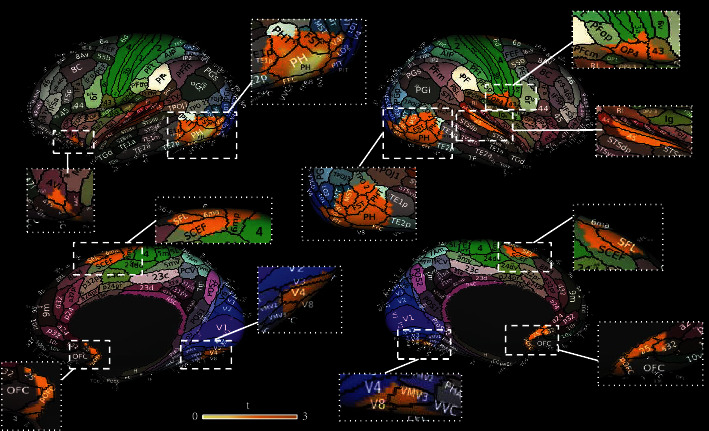
Anatomical mapping of off-target results. Qualitative overlap between the Glasser Atlas and the BOLD changes outside the stimulated areas (orange) shows changes in the primary and secondary visual areas, auditory cortex, subgenual cingulate cortex, and SMA.

Figure 5 shows the overlap between BOLD changes at whole brain level and the Glasser Atlas, highlighting the modulation in primary visual areas (V8, V4, and V3) as well as in visual areas (middle temporal area (MT), medial superior temporal area (MST), fourth visual area (V4te), fundal superior temporal areas (FST), and parahippocampal area (PH)), auditory cortex (A5), subgenual cingulate cortex (pOFC, area 25), and SMA (6ma, SFL), showing off-target effects of 40 Hz tACS. Whereas the primary visual cortices process visual information, the extrastriate visual areas (MT, MST) are considered hubs for the motion perception, the integration of local motion signals into global percepts, and guidance of eye movements [65], suggesting a potential role of tACS-induced phosphenes on these activations. For a more comprehensive insight on the anatomical and functional aspects of each region, refer to Glasser et al.

**Figure 6:**
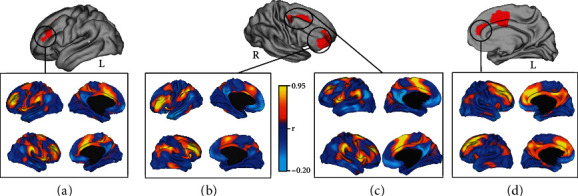
Network mapping of primary and secondary on-target results. (a, b) Show the network mapping of the clusters' peak in the prefrontal cortices. The regions correspond to the left and right dorsolateral prefrontal cortex (MNI coordinates (*x*, *y*, *z*): 34, 34, and 18; -30, 32, and 26). The mapping of the clusters' peak in the premotor cortex (MNI coordinates (*x*, *y*, *z*): 14, 6, and 64) and ACC (MNI coordinates (*x*, *y*, *z*): -20, 22, and 34) are reported in (c) and (d), respectively. R: right; L: left.

**Figure 7:**
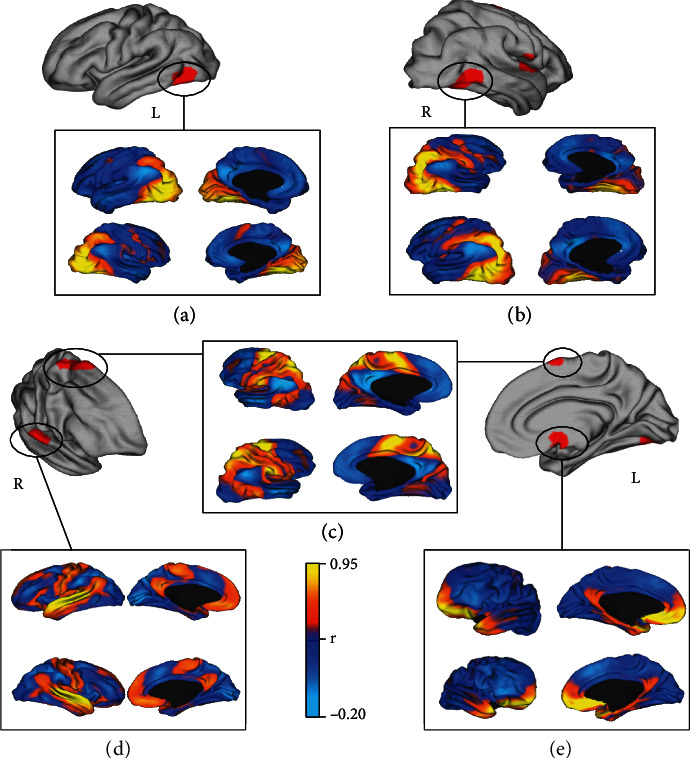
Network mapping of off-targets results. The figure shows the functional connectivity of the bilateral occipital cortices (a, b), corresponding to the right and left fusiform gyrus (MNI coordinates (*x*, *y*, *z*): -36, -70, and -18; 56, -58, and -14), of the supplementary motor area (c) (MNI coordinates (*x*, *y*, *z*): 14, 6, and 64); of the superior temporal gyrus (d) (MNI coordinates (*x*, *y*, *z*): 66, -8, and 4) and of the subgenual cortex (e) (MNI coordinates (*x*, *y*, *z*): 4, 4, and -24). R: right; L: left.

Figures 6 and 7 show the results of the seed-to-voxel analysis ran on a database of 1000 healthy participants [57] using the Neurosynth software. The functional connectivity of the primary on-target results (DLPFC) resembles both the Dorsal Attention Network (DAN) and the Anterior Salience Network (AS) (Figures 6(a) and 6(b)). Similarly, also, the functional connectivity computed on the secondary on-target results (premotor cortex and ACC) resembles both the AS (Figures 6(c) and 6(d)), with a more spread activity in the prefrontal cortices considering ACC functional connectivity (Figure 6(d)).

The functional connectivity of the off-target results has been computed on occipital lobes, SMA, temporal lobe, and subgenual cortex, as shown in Figure 7. Similarly to DLPFC, occipital lobes' functional connectivity resembles the DAN (Figures 7(a) and 7(b)). This network is particularly involved in generating and maintaining endogenous attention sets by a top-down cognitive selection of stimuli and comprises functionally connected brain regions including visual motion area. On the other hand, AS contributes to a variety of complex brain functions, and it is considered a dynamic hub for detection and selection of salient stimuli and for mediating interactions with other neurocognitive systems [66–68]. Moreover, also, the functional connectivity of SMA mostly resembles the DAN and the Sensorimotor Network (SMN) (Figure 7(c)), whereas the temporal cluster reveals a functional connection to Sensorimotor and Auditory Networks (Figure 7(d)). Finally, the seed-to-voxel analysis on the subgenual cortex does not show a clear network, but a spread functional connectivity over the orbitofrontal cortex and the temporal poles (Figure 7(e)).

## 4. Discussion

Considering the involvement of *γ* frequency in multiple cognitive functions as well as in neurological and psychiatric disorders (e.g., Alzheimer and Parkinson disease, schizophrenia, and frontotemporal dementia), several studies have evaluated the possibility to modulate cognitive performance through *γ*-tACS applied over the DLPFC (reviewed in [35]). We applied 40 Hz tACS over the bilateral DLPFC during fMRI, showing preliminary results of both on- and off-target effects, including activations involving the visual cortices, SMA, and subgenual cortex. The study, based on a block design of subsequent on and off stimulation periods, was not aimed at deciphering after-effects of tACS, but rather at disclosing local and network effects of the stimulation delivered at 40 Hz, a step of knowledge that is still lacking in the available scientific literature. A discussion on the specific findings and implications is provided below, as well as their limitations and possible future directions.

### 4.1. On-Target Effects of 40 Hz tACS

Although there is evidence about the efficacy of 40 Hz tACS in cognitive enhancement, especially in high-level functions such as working memory, attention, memory, and motor learning (for a comprehensive review, see [69]), very little evidence on the impact of 40 Hz tACS on brain dynamics is available. In this study, we showed online BOLD signal changes during 40 Hz tACS applied over the bilateral DLPFC in the regions corresponding to the normal electric field modelling, a result that seems further confirmed by the functional connectivity analysis (Figure S1). Moreover, a modulation of BOLD activity has been also found outside the stimulation target, in the posterior part of DLPFC (BA8), the ACC, and the right premotor cortex as shown in Figure 4. Even though these regions were not targeted directly by the tACS, they were likely reached by stimulation according to biophysical modelling (Figure 1(e)). These results, even if in line with modelling work, are partially at odds with previous resting-state fMRI-tACS studies that have never reported BOLD changes directly under the stimulation electrodes. Recently, Gundlach and colleagues [70] reported a modulation in the Eigenvector Centrality Measures (ECM) in the left primary somatosensory cortex (S1) during the application of 10 Hz tACS over bilateral S1, whereas no significant results under the electrode were detected when 65 Hz tACS was applied [70]. However, the authors found increases in ECM of the right DLPFC, as well as an increase in connectivity between a seed in S1 and the insula, cerebellum, left temporal gyrus, left precentral, and postcentral gyri during 65 Hz tACS as compared to sham. The increased focality and higher current density of the relatively smaller electrodes used in our study could potentially explain the observed effect on the DLPFC target, but further studies investigating the impact of tACS on local dynamics, including hemodynamic ones measured via Arterial Spin Labeling (ASL), are needed.

On the other hand, connectivity changes in brain regions not directly stimulated have been reported by Cabral-Calderin and colleagues [50, 51] when applying tACS to the occipital cortices. The authors revealed that tACS effects are not limited to regions below the electrodes but are influenced by networks' interactions, thus modulating mostly internetwork functional connectivity, while intranetwork functional connectivity changes are modest [50]. The seed-to-voxel analysis performed in our study is in line with this notion, showing changes in brain regions functionally connected to DLPFC (BA8 and ACC). The possible modulation of ACC by stimulating the left DLPFC has been already proved using another neuromodulatory technique, the repetitive Transcranial Magnetic Stimulation (rTMS), showing ACC-DLPFC connectivity links as targets for effective treatment for depression [71, 72]. Even though the mechanisms of neural activation of TMS and tES are profoundly different, these studies provided strong evidence that stimulation of left DLPFC may influence the ACC, possibly based on their shared connectivity profile, also opening to the possibility to modulate distant—possibly deep—brain regions trough NIBS.

Observed local, as well as network-like, effects of 40 Hz tACS could be beneficial in psychiatric disorders like schizophrenia [25, 73] and autism [74, 75], as well as in neurodegenerative disorder as Alzheimer's disease (AD) [76, 77], characterized by a dysregulation of oscillatory activity in the gamma frequency and a shift from faster (e.g., gamma) to slower (e.g., theta) brain activity [10]. For example, clinical potential of restoring *γ* oscillations through alternating current stimulation has been proved in a mouse model of Alzheimer's dementia (AD). In particular, animal studies have revealed that the induction of *γ* frequency activity through sensory stimulation or optogenetics reduces amyloid-*β* plaques [78, 79], as well as that optogenetic modulation of parvalbulmin (PV+) and somatostatin (SST+) interneurons restores *γ* oscillations in murine models of AD [80]. Preliminary evidences are also arising from pilot studies using 40 Hz tACS in AD patients [81, 82].

### 4.2. Off-Target Effects of 40 Hz tACS

Previous studies aimed at modulating functional connectivity via *γ*-tACS have already demonstrated that stimulation effects can reverberate also in brain regions distant from the targets but still functionally connected to them, as explained in the previous paragraph [50, 51]. Our results showing the modulation of SMA by stimulating DLPFC could also have been driven by the functional connection between the two areas. Several studies have shown that DLPFC and SMA are both associated with cognitive processes related to attention and executive functions initiated by the frontal areas [83]. Moreover, SMA is part of the motor systems and plays a significant role in movement planning, control, and execution [84]. Therefore, an increased metabolic activity in SMA and DLPFC may imply an increase in receiving, processing, and integrating visual and motor signals to guide ongoing behavior, enhancing the top-down integration of DLPFC to the motor cortex.

However, the whole-brain analysis revealed off-target effects also in visual cortices, as well as in subgenual and temporal areas, usually not functionally linked to DLPFC and neither predicted by the biophysical modelling. Several EEG studies suggested that responses in the visual cortices could be caused by the tACS-evoked phosphenes: for example, 6 Hz photic stimulation produces phase-locked EEG driving responses in the 6, 12, and 18 Hz frequency range [85]. Further support to this idea comes from fMRI studies showing a bilateral activation of the thalamus and DLPFC in response to photic stimulation [86, 87], providing a possible pathway for retinal phosphenes to influence brain activity also in nonvisual areas. Additionally, several studies showed that tACS current spread can evoke retinal phosphenes [46–48], especially when the tACS electrodes are placed closer to the eyes [46, 47, 88, 89]. Off-target effects of 40 Hz tACS were also found over the extrastriate cortices (middle temporal (MT) and medial superior temporal (MST) areas). Originally discovered in the macaques' brain, MT and MST areas are considered crucial hubs for visual motion processing also in human, consequently suggesting their activation could potentially be caused by retinal phosphenes [90, 91]. Besides the possible entrainment effects induced by retinal phosphenes in the primary and extrastriate visual cortices in our study, the perception of phosphenes may also have modified the alertness of participants [92]. The functional connectivity mapping performed in the corresponding clusters of activation has indeed revealed similarity within these nodes and the Dorsal Attention Network (DAN). This network is known to be activated by a task that required attention and allows the selection of sensory stimuli based on internal goals (goal-driven attention), linking them to motor responses. Therefore, we cannot not rule out that the BOLD signal changes on the primary and extrastriate visual cortices are associated to the phosphene phenomenon. Future studies should address this specific aspect via *ad hoc* control conditions.

The correlation between the DLPFC and subgenual cortex has been extensively investigated in studies aimed at predicting the effect of NIBS treatment in patients with depression [93, 94]. Functional connectivity between these two nodes is helpful in differentiating patients with depression from healthy controls and in predicting TMS treatment results [95]. In healthy subjects, the link between DLPFC and subgenual cortex is characterized by a mutual inhibition process: the activation of DLPFC during a task is associated with inhibition of limbic regions, including the subgenual cortex, and vice versa [96]. Interestingly, negative correlation in the EEG *γ* band between subgenual cortex and left DLPFC has been recently observed [97]. However, the increased BOLD activity in both areas reported here could have a twofold explanation: (i) the higher activation of DLPFC during 40 Hz tACS drives the activation of subgenual cortex through their well-known functional connectivity; (ii) the subgenual cortex increased its activation in an attempt to suppress the 40 Hz tACS induced higher activation of DLPFC, following their inhibitory interplay.

### 4.3. Limitations of the Study and Future Directions

The first limitation of our study is the exploratory nature of the work that does not include a sham condition. However, a recent study showed that short stimulation periods did not produce after-effects in amplitude or phase of the EEG signals [98]. Even though we could not generalize our findings to the results obtained by Strüber and colleagues considering the difference in the stimulation's duration (1 vs. 60 seconds), we speculated that 60 seconds of tACS could not be enough to produce after-effects. This is also corroborated by the lack of BOLD modulation in the off blocks when compared to on blocks, indicating a possible absence of after-effect of tACS if applied for short periods, even though this result is going beyond our goals. Recently, Pozdniakov and colleagues (2021) also demonstrated that neither the 10 Hz nor the 20 Hz of 15 minutes of stimulation induced tACS offline effects, when the stimulation target was the motor cortex [99]. Moreover, Pahor and Jaušovec revealed that gamma tACS had no significant effect on EEG amplitude following 15 min of sham or active tACS in any of the frequency bands of interest [15].

Future studies with electrophysiological recordings are needed in order to specifically evaluate the after-effect of 40 Hz tACS.

Another limitation of our exploratory study is the lack of a control condition using another stimulation frequency. This would have allowed a clear estimation of frequency specificity of the effects. An experimentally controlled parametric modulation of the target stimulation frequency would be desirable for future studies. Moreover, stimulation effects do not only depend on the target but also depend on the interaction of the specific stimulation frequency with endogenous neural frequencies [100–103]. Stimulating at frequencies not aligning to the endogenous oscillation frequency might decrease the effectiveness of the entraining, thus making the targeting of functionally relevant endogenous rhythms a crucial aspect to improve the spatial and functional specificity of tACS [104, 105]. Consequently, future tACS studies may benefit from closed-loop system where tACS frequencies are dynamically adjusted based on endogenous or task-driven oscillations [106].

In the present study, we used only one electrode montage. Due to the explorative nature of the study, we opted for the most used target in clinical and experimental research (F3-F4), promoting generalization and applicability of results to several currently used protocols. In addition, computational model considering the location of electrodes as well as the stimulation phase could provide a useful tool for guiding electrode placement for future tACS studies.

A final limitation is the absence of behavioral tasks testing the functional relevance of the observed BOLD changes. This limitation is intrinsic to the experimental questions that were based on very short stimulation periods and assumed no after-effects.

## 5. Conclusion

Current results expand the evidence on online effects of gamma-band tACS applied during resting-state condition on bilateral DLPFC, providing relevant details on modelling-based target engagement and network-level effects. Findings might help the design of future interventions in both healthy individuals and psychiatric and neurological disorders characterized by a dysregulation of gamma activity.

## Figures and Tables

**Table 1:** MNI coordinates for the main regions showing the increased BOLD signal in the on > off contrast masked for normE-field.

Activation loci	Cluster size	*p* (unc)	Peak *T* value	Peak coordinates (MNI)		
				*x*	*y*	*z*
Premotor cortex	202	0.0024	7.242	14	6	64
			6.349	20	0	62
			4.921	2	6	62

Right dorsolateral prefrontal cortex	181	0.0037	5.031	34	34	18
			4.077	28	38	26
			3.991	18	36	36

Anterior cingulate cortex	188	0.0032	4.549	-20	22	34
			4.475	-26	26	30

Left dorsolateral prefrontal cortex	156	<0.0001	4.062	-30	32	26
			4.003	-40	26	42

**Table 2:** MNI coordinates for the main regions showing the increased BOLD signal in the on > off contrast at whole brain level.

Activation loci	Cluster size	*p* (FWE-corrected)	Peak *T*	Peaks coordinates (MNI)		
				*x*	*y*	*z*
Subgenual cortex	509	<0.0001	10.13	4	4	-24
			9.53	-6	4	-24

Right visual cortex	274	<0.0001	8.18	56	-58	-14
			6.23	40	-70	-18

Right temporal gyrus	126	<0.0001	7.99	66	-8	-4

Left visual cortex	181	<0.0001	7.27	-36	-70	-18
			5.23	-48	-64	-14

Supplementary motor cortex	347	<0.0001	7.24	14	6	64
			6.64	22	0	60

## Data Availability

Raw data and codes will be available upon request to E.S.

## Supplementary Materials

Supplementary Materials Supplementary Information: Methods; Results; Supplementary Figures 1 and 2.
