# Supplementary material for: Local and Distributed fMRI Changes Induced by 40 Hz Gamma tACS of the Bilateral Dorsolateral Prefrontal Cortex: A Pilot Study
Source: Neural Plast. 2022 Jul 16;2022:6197505. doi: 10.1155/2022/6197505 (PMC9308536; doi:10.1155/2022/6197505)
Supplement: Supplementary Materials — Supplementary Information: Methods; Results; Supplementary Figures 1 and 2. [file 6197505.f1.docx]

**Local and distributed fMRI changes induced by 40Hz gamma tACS**

**of the bilateral dorsolateral prefrontal cortex: a pilot study**

Mencarelli Lucia^1,2^, PhD, Monti Lucia^3^, MD, Romanella Sara^1^, MS, Neri Francesco^1^, MS, Giacomo Koch^2^, MD, Salvador Ricardo^4^, PhD, Ruffini Giulio^4^, PhD, Sprugnoli Giulia^1^, MD, Rossi Simone^1,5^, MD, PhD, Santarnecchi Emiliano^6^, PhD, PsyD

1 Siena Brain Investigation & Neuromodulation Lab (Si-BIN Lab), Department of Medicine, Surgery and Neuroscience, Neurology and Clinical Neurophysiology Section, University of Siena, Italy

^2^ Non-invasive Brain Stimulation Unit, Department of Behavioral and Clinical Neurology, Santa Lucia Foundation IRCCS, Rome, Italy

^3^ Unit of Neuroimaging and Neurointervention, “Santa Maria alle Scotte” Medical Center, Siena, Italy

4 Neuroelectrics, Cambridge, MA (USA) and Barcelona (Spain)

5 Human Physiology Section, Department of Medicine, Surgery and Neuroscience, University of Siena, Siena, Italy

6 Precision Neuromodulation Program & Network Control Laboratory, Gordon Center for Medical Imaging, Department of Radiology, Massachusetts General Hospital, Harvard Medical School, Boston, MA, USA

**Supplementary Information**

**Corresponding author:**

Emiliano Santarnecchi, PhD, Psy

Precision Neuromodulation Program & Network Control Laboratory,

Gordon Center for Medical Imaging, Department of Radiology, Massachusetts General Hospital,

Harvard Medical School, Boston, MA, USA

office +1-617-667-0326; mobile +1-617-516-9516

[esantarnecchi@mgh.harvard.edu](mailto:esantarnecchi@mgh.harvard.edu)

**METHODS**

***Functional Connectivity Analysis***

In order to explore whether the 40Hz-tACS induced effects also in term of functional connectivity, we performed a seed-to-voxel analysis considering as seeds both left and right DLPFC (i.e., the stimulated regions). Temporal correlations were computed between these seeds and all other voxels in the brain. Statistical analysis was carried out using the CONN (v.20b) toolbox and Matlab 2018b software (Mathworks, MA, USA). A two-tailed t-test was conducted comparing the blocks ON to the blocks OFF; age and gender were included as covariates in the analysis. Results were computed applying a cluster size correction (p< 0.05, false discovery rate -FDR- corrected).

**RESULTS**

***Functional Connectivity Changes***

In line with BOLD analysis, the seed-to-voxel analysis revealed a significantly stronger connectivity between the left and right DLPFC and a large cluster of voxels in the frontal regions, including right and left Superior Frontal Gyrus, left Middle Frontal Gyrus, left Inferior Frontal Gyrus, and right and left Frontal Pole (*k*=1119; -14, 40, 38; Figure S1). Thus, connectivity changes seem to follow the topography of the induced E-field (Figure 1E).


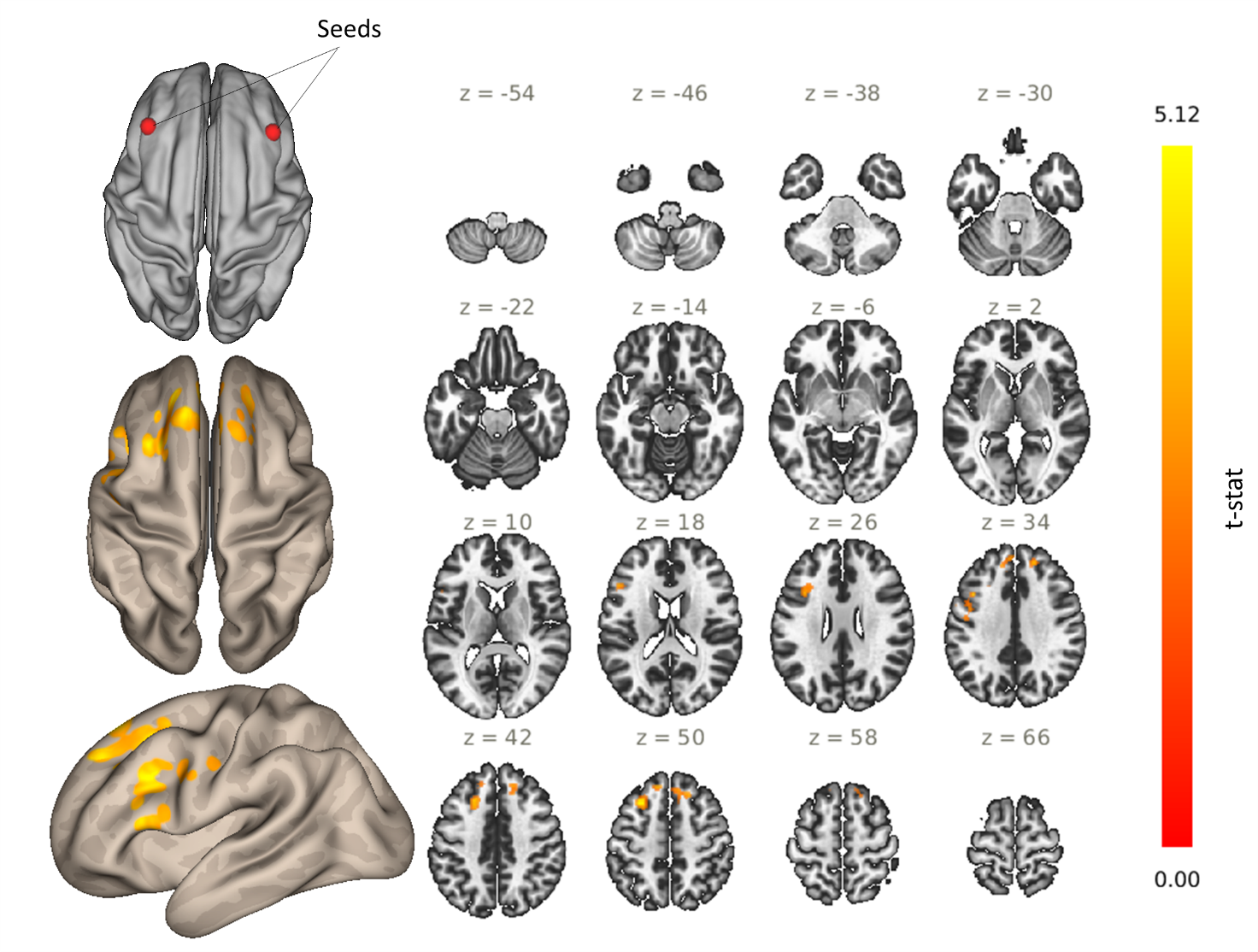


**Figure S1. Seed-to-voxel analysis.** Blocks contrasts of seed-to-voxel connectivity maps considering left and right DLPFC as seeds showing significant (p<0.05 FDR-corrected) increased functional connectivity with frontal areas. The colorbar represents the t-statistic. Images are presented in neurological convention.


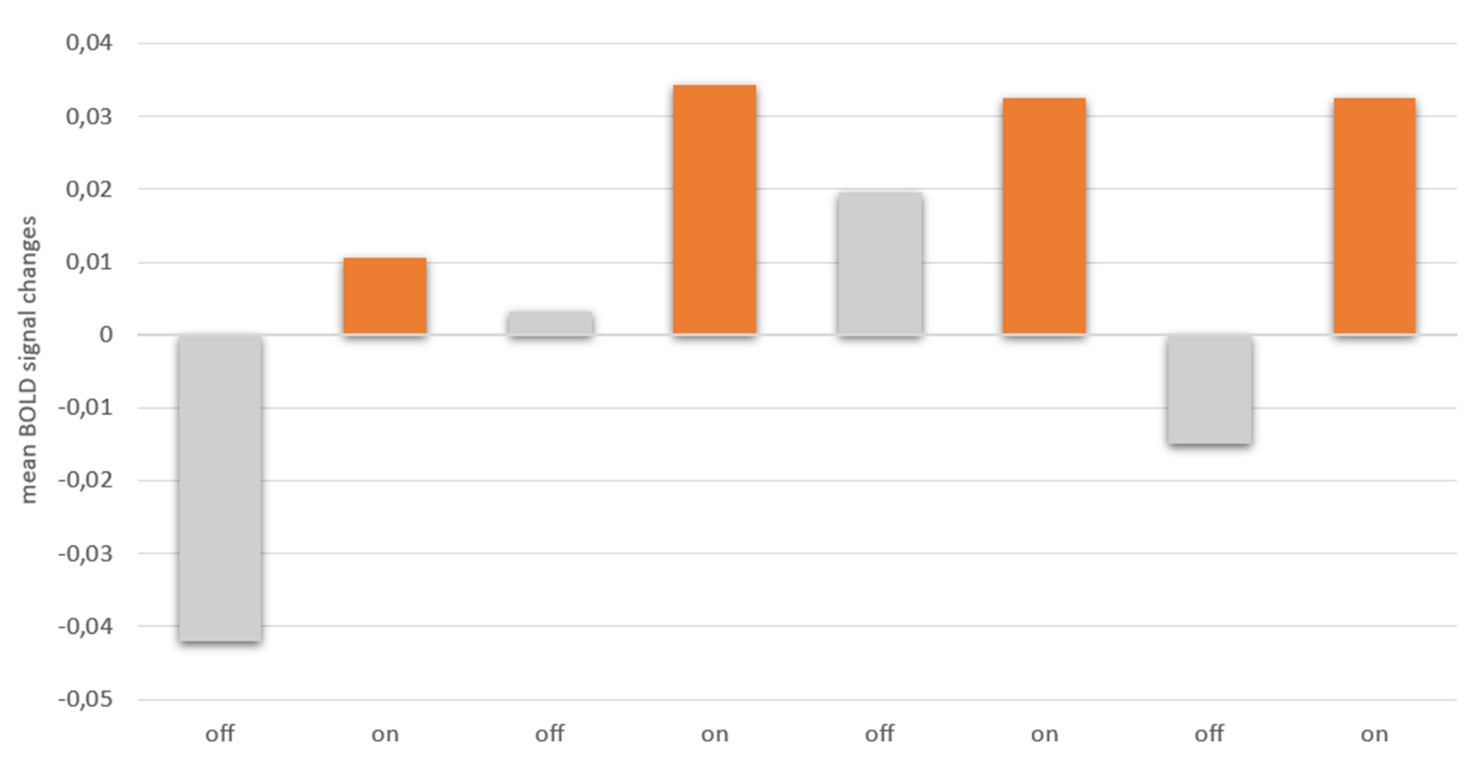


**Figure S2. Frontal BOLD changes.** Mean of the BOLD signal changes extracted from two frontal ROIs (r= 10 mm) centered around the activation peak (rDLPFC: 34, 34, 18; lDLPFC: -30, 32, 26). During the ON blocks the BOLD signal showed an initial increase in the first block followed by higher increments during the following ones remaining stable over time. On contrary, the BOLD signal during the OFF blocks showed an unstable and variegated pattern of changes. Grey= OFF; Orange= ON.
